# Lighter serum copper isotopic composition in patients with early non-alcoholic fatty liver disease

**DOI:** 10.1186/s13104-020-05069-3

**Published:** 2020-04-19

**Authors:** Sanne Van Campenhout, Agustina A. M. B. Hastuti, Sander Lefere, Hans Van Vlierberghe, Frank Vanhaecke, Marta Costas-Rodríguez, Lindsey Devisscher

**Affiliations:** 1 Department of Internal Medicine and Pediatrics–Hepatology Research Unit, Ghent University, Corneel Heymanslaan 10 entrance 12-floor 1, 9000 Ghent, Belgium; 2 Department of Basic and Applied Medical Sciences–Gut Liver Immunopharmacology Unit, Ghent University, Corneel Heymanslaan 10 entrance 36–floor 3, 9000 Ghent, Belgium; 3 Department of Chemistry, Atomic and Mass Spectrometry–A&MS Research Unit, Ghent University, Campus Sterre, Krijgslaan 281-S12, 9000 Ghent, Belgium

**Keywords:** Liver steatosis, Non-alcoholic steatohepatitis, Cu isotopes, Multi collector inductively coupled plasma mass spectrometry

## Abstract

**Objective:**

The occurrence of non-alcoholic fatty liver disease (NAFLD) is globally increasing. To challenge the current incidence of NAFLD, non-invasive markers that could identify patients at risk or monitor disease progression are an important need. Copper intake and organ copper concentrations have earlier been linked to NAFLD progression, but serum copper does not adequately represent the disease state. Cu atoms occur under the form of two stable isotopes, ^63^Cu and ^65^Cu, and the ratio of both (expressed as δ^65^Cu, in  ‰) in blood serum has been shown to be altered in chronic liver disease. To assess whether the Cu isotope ratio might predict disease occurrence and progression of NAFLD, the serum Cu isotopic composition of patients with different stages of NAFLD was determined.

**Results:**

Our results showed that serum δ^65^Cu values were lower in NAFLD patients, already at the level of simple steatosis, and remained stable during further disease progression. ROC analysis shows an almost perfect diagnostic ability of serum δ^65^Cu values for NAFLD, but no discrimination between different severity degrees could be made. Therefore, the serum Cu isotopic composition might show potential for early diagnosis of NAFLD patients.

## Introduction

Non-alcoholic fatty liver disease (NAFLD) is the most common liver disease with a global current prevalence of 24% [1]. In the past two decades, its incidence has increased about fivefold, especially in young adults [2]. NAFLD can range from simple hepatic steatosis (fat accumulation, non-alcoholic fatty liver, NAFL) to liver inflammation (non-alcoholic steatohepatitis, NASH), which can evolve to liver fibrosis with varying degree of liver dysfunction [3]. Since patients with steatohepatitis are at increased risk of fibrosis development and might even develop hepatocellular carcinoma (HCC), and no pharmacological option for any stage of NAFLD is available so far, it is of interest to detect patients early in disease progression.

Recently, there has been a growing interest in high-precision Cu isotopic analysis, i.e. evaluating the ^65^Cu/^63^Cu ratio (expressed as δ^65^Cu, in  ‰), as a diagnostic and prognostic tool for liver disease [4–7]. ^63^Cu and ^65^Cu are the two stable Cu isotopes that occur naturally, showing small, but systematic variations in their abundance ratio under certain disease conditions that can be reliably quantified [8–10]. It has been published recently that patients destined for bariatric surgery may show lower serum δ^65^Cu values [11]. Bariatric surgery is performed on morbidly obese patients to reduce food intake and obtain weight loss, and NAFLD is a prominent health risk in these patients. In the latter study, no liver biopsies were taken and no fibroscan was used to assess NAFLD in this patient cohort, and thus the initial status of liver disease was not known.

Therefore, we have investigated the diagnostic potential of serum δ^65^Cu values for different stages of NAFLD. We have collected serum from patients with biopsy-proven NAFLD with varying severity, and subjected these samples to high-precision Cu isotopic analysis via multi-collector inductively coupled plasma-mass spectrometry (MC-ICP-MS).

## Main text

### Materials and methods

#### Patient cohort

A patient cohort of 10 NAFL and 14 NASH patients was recruited at the Ghent University Hospital within the context of a previous study (Table 1 for patient characteristics). Non-cirrhotic patients were scheduled for bariatric surgery. Specific clinical, biochemical, histological and/or radiographic criteria were used to exclude liver disease of other aetiologies, including cholestatic, alcohol-induced or drug-induced liver disease and viral or auto-immune hepatitis. All patients had biopsy-confirmed NAFLD and a negative history of alcohol abuse (average daily consumption of less than 20 g EtOH). Healthy controls from previous studies were used for comparison (Table 1) [11–13].

**Table 1:** Clinical characteristics of the patient cohort. Results are expressed as mean ± 95% CI

Characteristic	NAFL (n = 10)	NASH F0-F2 (n = 10)	NASH F4 (n = 4)
Age (years)	39.8 ± 7.8	47.5 ± 8.9	66.8 ± 17.1
Gender (M %/F %)	10/90	0/100	100/0
BMI (kg m^−2^)	41.6 ± 6.5	42.7 ± 7.2	29.0 ± 11.2
Type 2 diabetes (%)	10	30	100
AST (U l^−1^)	23.3 ± 4.2	32.6 ± 17.0	31.8 ± 21.5
ALT (U l^−1^)	24.2 ± 5.4	39.4 ± 24.3	33.3 ± 11.7
GGT (U l^−1^)	16.0 ± 4.2	51.5 ± 27.8	139.5 ± 142.6
Platelets (10^3^ µl^−1^)	274.5 ± 29.9	297.3 ± 64.1	133.8 ± 87.0
Hb (g dl^−1^)	13.2 ± 0.6	13.5 ± 1.3	12.4 ± 3.4
HbA1c (%)	5.6 ± 0.3	6.1 ± 1.2	6.2 ± 1.8
Total cholesterol (mg dl^−1^)	186.8 ± 21.1	195.4 ± 40.0	
LDL (mg dl^−1^)	94.2 ± 16.3	103.2 ± 21.0	
HDL (mg dl^−1^)	66.3 ± 11.8	44.4 ± 13.0	
Triglycerides (mg dl^−1^)	121.7 ± 26.7	219.9 ± 76.5	
Bilirubin (mg dl^−1^)	0.4 ± 0.1	0.4 ± 0.1	
Fasting glucose (mg dl^−1^)	98.2 ± 18.5	97.5 ± 9.6	
HOMA-IR	4.4 ± 2.4	6.9 ± 2.9	
Iron (µg dl^−1^)	76.6 ± 26.4	66.3 ± 19.0	
Transferrin (mg dl^−1^)	2.9 ± 0.3	2.6 ± 0.7	
Ferritin (µg l^−1^)	60.0 ± 38.6	155.1 ± 122.3	
Copper (µg dl^−1^)	137.4 ± 38.4	117.3 ± 39.6	

Control population			
REF	Age, years	Gender (M%/F%)	n
[11]	43.8 ± 8.5	0/100	10
[12]	23–48	100/0	5
[13]	51.57 ± 6.9	100/0	7

*NAFL* non-alcoholic fatty liver, *NASH* non-alcoholic steatohepatitis, *BMI* body mass index, *AST* aspartate aminotransferase, *ALT* alanine aminotransferase, *GGT* γ-glutamyltransferase, *Hb* Haemoglobin, *HbA1c* haemoglobin A1c, *LDL* low-density lipoprotein, *HDL* high-density lipoprotein, *HOMA-IR* Homeostasis Model Assessment Insulin Resistance

#### Clinical evaluation

Anthropometric measurements of all patients were taken and the body mass index (BMI) was calculated as body weight/height^2^ (kg m^−2^). Blood samples from patients were collected, centrifuged (10 min, 4 °C, 350 g), and serum was stored at − 80 °C until analysis. Thawed serum was analysed for the levels of aspartate aminotransferase (AST), alanine aminotransferase (ALT), γ-glutamyl transpeptidase (GGT), bilirubin, haemoglobin, triglycerides, high-density lipoprotein (HDL) cholesterol, low-density lipoprotein (LDL) cholesterol, total serum cholesterol, fasting glucose, haemoglobin A1c (HbA1c) and insulin and complete blood count. Insulin resistance was estimated by the homeostasis model assessment-insulin resistance (HOMA-IR) [14], and the presence of diabetes mellitus (according to the American Diabetes Association criteria [15]) was evaluated. In addition, iron, transferrin, ferritin and copper serum levels were determined.

Formalin-fixed liver biopsies were routinely processed and stained with haematoxylin and eosin, and Masson’s trichrome. All biopsies were blinded and evaluated by an experienced pathologist according to the NASH clinical research network scoring system [16].

#### Serum isotopic analysis

Serum samples (400 µL), were thawed and digested in perfluoroalkoxy (PFA) digestion vessels (Savillex, USA) with a 2:1 v/v mixture of 14 M *pro analysis* HNO_3_ (Chem-lab, Belgium and additionally purified by means of sub-boiling distillation) and 9.8 M H_2_O_2_ (Sigma-Aldrich, Belgium). The digestion was performed at 110 °C for 18 h. The digest was then dried at 90 °C and the solid residue was re-dissolved in 5 mL of 8 M *Optima™* grade HCl (Fisher Chemical, UK) and 0.001% H_2_O_2_ to ensure that Cu is present in its Cu(II) oxidation state.

Cu was isolated by anion exchange chromatography using 1 mL of AG-MP-1 resin (Bio-Rad Laboratories, CA, USA) [12]. Two column passes ensure sufficient purity, while a quantitative recovery avoids potential on-column isotope fractionation effects from affecting the final Cu isotope ratio results. The purified Cu fractions thus obtained were dried at 90 °C and the residue was re-dissolved in 1 mL of 14 M HNO_3_. This was done twice to fully remove remaining chlorine. Finally, the Cu fractions were re-dissolved in 0.42 M HNO_3_ for further elemental and isotopic analysis. Ultra-pure water (resistivity > 18.2 MΩ cm) was used throughout this study (Milli-Q Element water purification system, Millipore, France). All sample manipulations were performed in a class-10 clean room.

Cu isotope ratio measurements were performed using a Neptune MC-ICP-MS instrument (Thermo Scientific, Germany), as described elsewhere [5]. Samples were measured in sample-standard bracketing (SSB) sequence using a solution of NIST SRM 976 Cu isotopic reference material (NIST, USA) as external standard. Cu concentrations in samples and standard were matched within ± 10%. The mass bias was corrected for using a combination of internal standardisation using Ga as internal standard, relying on the revised Russell’s law as described by Baxter et al. [17], and external correction in an SSB approach. The Cu isotope ratio is expressed in delta notation as δ^65^Cu (‰), calculated as indicated in Eq. 1:

1 $$\delta^{65} Cu_{sample} = \left( {\frac{{{{{}^{65}Cu} \mathord{\left/ {\vphantom {{{}^{65}Cu} {{}^{63}Cu}}} \right. \kern-0pt} {{}^{63}Cu}}_{sample} }}{{{{{}^{65}Cu} \mathord{\left/ {\vphantom {{{}^{65}Cu} {{}^{63}Cu}}} \right. \kern-0pt} {{}^{63}Cu}}_{{NIST^{{}} SRM^{{}} 976}} }} - 1} \right) \times 1000$$

A Cu in-house standard (Inorganic Ventures, the Netherlands) was measured every five samples to monitor the quality of the isotope ratio data. The Cu isotope ratio obtained for the in-house QC sample in this work was δ^65^Cu = 0.223 ± 0.009 (sd, n = 9), which corresponds well with the value obtained in our previous study [12].

#### Statistics

Statistical analysis was performed using Graphpad Prism (Graphpad Software Inc., San Diego, California) and SPSS Statistics 25 (IBM Corp., USA). Kruskall-Wallis and Dunn’s multiple comparisons test were used to compare groups and Receiver Operating Characteristic (ROC) analysis was performed. Spearman’s rank correlation coefficient was used to evaluate potential relationships between biochemical parameters and the Cu isotopic composition within the NAFLD population. P-values were two-sided and considered significant when lower than 0.05.

### Results

**Fig. 1:**
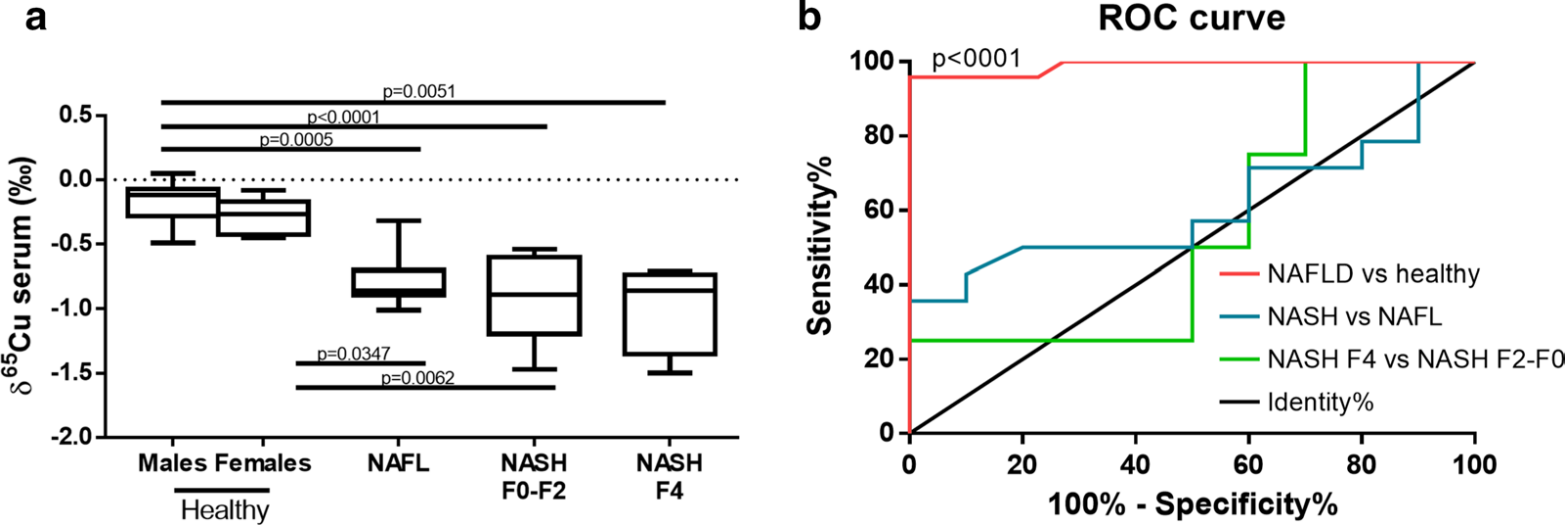
Serum δ^65^Cu changes in non-alcoholic fatty liver disease (NAFLD). **a** Serum δ^65^Cu values for different stages of NAFLD. Statistical analysis was performed by Kruskal–Wallis and Dunn’s multiple comparisons test. **b** Receiver Operating Characteristic (ROC) analysis of serum δ^65^Cu values. P-values were two-sided and considered significant when lower than 0.05. F0–F4 refers to the corresponding fibrosis stage. Healthy males: n = 12, healthy females: n = 10. Non-alcoholic fatty liver (NAFL): n = 10. Non-alcoholic steatohepatitis (NASH) F0–F2: n = 10. NASH F4: n = 4

In NAFLD patients, serum δ^65^Cu values were lower than in healthy controls (Fig. 1a), even at the level of simple steatosis (NAFL) (p ≤ 0.04), and remained stable during further disease progression (Fig. 1a). More specifically, average serum δ^65^Cu values were lower by 0.57‰ ± 0.35‰ (sd), 0.71 ± 0.47‰ (sd), and 0.76 ± 0.51‰ (sd) in NAFL patients, NASH patients with F0–F2 fibrosis, and NASH patients with cirrhosis (F4), respectively. This suggests that the lower serum δ^65^Cu value is an early event in NAFLD and is thus already present before the onset of liver inflammation.

Importantly, ROC analysis shows an almost complete (AUC = 0.9896, p < 0.0001) diagnostic ability of serum δ^65^Cu values for NAFLD patients (Fig. 1b). In contrast, no further discrimination can be made between NAFL and NASH patients (AUC = 0.6107, p = 0.36) or between NASH patients with F0–F2 fibrosis and with cirrhosis (F4) (AUC = 0.5500, p = 0.78). In addition, no relevant or significant correlations between clinical parameters and serum δ^65^Cu values within the NAFLD patient cohort were observed (max |ρ| = 0.22, Table 2).

**Table 2:** Spearman’s correlation analysis between serum δ^65^Cu levels and clinical parameters within the NAFLD patient cohort

	Spearman’s ρ	p	n
BMI (kg m^−2^)	0.03	0.89	24
Steatosis on liver biopsy	− 0.22	0.34	21
Ballooning on liver biopsy	− 0.20	0.37	21
Inflammation on liver biopsy	− 0.09	0.71	21
NAS on liver biopsy	− 0.19	0.42	21
Fibrosis on liver biopsy	− 0.13	0.54	24
Presence of NASH (yes/no)	− 0.19	0.38	24
AST (U l^−1^)	0.25	0.23	24
ALT (U l^−1^)	0.03	0.89	24
GGT (U l^−1^)	− 0.05	0.81	24
Bilirubin (mg dl^−1^)	− 0.07	0.77	20
Glucose (mg dl^−1^)	0.24	0.30	20
HOMA-IR	0.26	0.27	20
HbA1c (%)	0.09	0.70	23
Diabetes (yes/no)	0.19	0.39	24
Cholesterol (mg dl^−1^)	0.22	0.39	17
HDL (mg dl^−1^)	− 0.10	0.71	17
LDL (mg dl^−1^)	− 0.06	0.80	19
Triglycerides (mg dl^−1^)	− 0.16	0.50	20
Ferritin (µg l^−1^)	0.24	0.33	19
Copper (µg dl^−1^)	0.10	0.69	18
Transferrin (mg dl^−1^)	− 0.03	0.91	19
Iron (µg dl^−1^)	− 0.03	0.92	19

*NAFL* non-alcoholic fatty liver, *NASH*: non-alcoholic steatohepatitis, *BMI*: body mass index, *AST* aspartate aminotransferase, *ALT* alanine aminotransferase, *GGT* γ-glutamyltransferase, *Hb* Haemoglobin, *HbA1c* haemoglobin A1c, *LDL* low-density lipoprotein, *HDL* high-density lipoprotein, *HOMA-IR* Homeostasis Model Assessment Insulin Resistance, *NAS* NAFLD activity score

### Discussion

Lower serum δ^65^Cu values have been described in patients with end stage liver disease (cirrhosis), arising from several aetiologies, in cancer patients, and in patients with Wilson’s disease [4–8]. In addition, we have shown a whole-body enrichment of the light ^63^Cu isotope in an experimental model for secondary biliary fibrosis [18]. Our data align those previous results and further elaborate on serum δ^65^Cu values within different NAFLD stages. In agreement with previously published data of a bariatric cohort [11], serum δ^65^Cu values were lower in NAFLD patients. The variation of serum δ^65^Cu values in different stages of NAFLD had not been investigated thus far, and we show that, already at the level of simple steatosis, serum δ^65^Cu values are lower, and remain stable during further disease progression. We highlight lower serum δ^65^Cu values as diagnostic tool for NAFLD, irrespective of disease progression. Currently, patients are screened for steatosis by non-invasive imaging techniques (such as ultrasound or the controlled attenuation parameter), which require patients to go to a hospital or center where this equipment is available [19]. In addition, the general practitioner can take blood samples for serum biomarker analysis (such as the fatty liver index or the SteatoTest). However, the diagnostic ability of these biomarkers for steatosis (AUROC ranging from 0.73–0.86) can still be improved [19]. Our results show potential for serum δ^65^Cu to exceed the diagnostic ability of current serum biomarkers, and to diagnose patients with early NAFLD, a population that would benefit from early notification to prevent progression to NASH, for which no medical therapy is available so far.

## Limitations

Although our patient population covers different stages of NAFLD, no obese patients without NAFLD were included. These patients could have revealed additional information about the initial stage and cause of the lower serum δ^65^Cu values. In addition, both genders were not equally represented in the different NAFLD stages. However, when we compare male and female healthy controls, the difference between average serum δ^65^Cu values was much smaller (0.12‰) than the observed disease effects (0.57–0.76‰) and not significant (Fig. 1a). A larger patient cohort could have also allowed to evaluate assay reproducibility and sensitivity. Nevertheless, serum δ^65^Cu values have previously been shown to be reproducible in healthy controls [11–13], so that these results seem highly reliable.

## Data Availability

The datasets used and/or analysed during the current study are available from the corresponding authors on reasonable request.

## Abbreviations

ALT: Alanine aminotransferase
AST: Aspartate aminotransferase
BMI: Body mass index
GGT: γ-Glutamyltransferase
Hb: Haemoglobin
HbA1c: Haemoglobin A1c
HCC: Hepatocellular carcinoma
HDL: High-density lipoprotein
HOMA-IR: Homeostasis Model Assessment Insulin Resistance
LDL: Low-density lipoprotein
NASH: Non-alcoholic steatohepatitis
NAFL: Non-alcoholic fatty liver
NAFLD: Non-alcoholic fatty liver disease
